# Supplementary material for: Predicting central choroidal thickness from colour fundus photographs using deep learning
Source: PLoS One. 2024 Mar 29;19(3):e0301467. doi: 10.1371/journal.pone.0301467 (PMC10980193; doi:10.1371/journal.pone.0301467)
Supplement: S1 File — (DOCX) [file pone.0301467.s001.docx]

**臨床研究等計画書**

1. 研究課題名　　　医用画像の機械学習用データベース構築

2. 研究の実施体制

2.1 実施体制の概要

□自治医科大学単独での研究

■自治医科大学を主管とする多機関共同研究

2.2 学内の実施体制

|  | 所　　属 | 職　名 | 氏　　名 | 役割及び責任 |
| --- | --- | --- | --- | --- |
| ①研究責任者 | 眼科 | 教授 | 川島　秀俊 | 統括・データ管理 |
| ②研究分担者 | 眼科 | 准教授 | 髙橋　秀徳 | 機械学習(解析・データ管理に関与せず) |
|  | 健診センター | 教授 | 宮下　洋 | 健診センターのデータ管理 |
|  | 眼科 | 講師 | 新井　悠介 | データ解析 |
|  | 眼科 | 助教 | 渡辺　芽里 | データ解析 |
|  | 眼科 | 助教 | 坂本　晋一 | データ解析 |
|  | 眼科 | 助教 | 伊野田　悟 | 機械学習 |
|  | 眼科 | 病院助教 | 粕谷　友香 | データ収集 |
|  | 眼科 | 病院助教 | 長岡　広祐 | データ収集 |
|  | 眼科 | 臨床助教 | 橋本悠人 | データ収集 |
|  | 眼科 | 臨床助教 | 吉田花 | データ収集 |
|  | 眼科 | 臨床助教 | 高山卓也 | データ収集 |
|  | 眼科 | フォトグラファー | 反保　宏信 | データ収集 |
| ③研究協力者 |  |  |  |  |
|  |  |  |  |  |

＜教育・研修の受講＞

- 研究責任者の受講状況等

倫理講習会：2021年07月09日

e-learning：

CREDITS【倫理・行動規範コース】2021年04月09日

CREDITS【臨床研究実施コース】2021年04月09日

■研究責任者は、研究分担者及び協力者の倫理講習会及びe-learningの受講を確認した。

2.3 共同研究機関の実施体制

| 機関名 | 所　属／職　名 | 氏　　名 | 役割及び責任 |
| --- | --- | --- | --- |
| さいたま医療センター眼科 | 教授 | 蕪城　俊克 | データ収集 |
| 青木眼科医院 | 院長 | 青木真祐※ | データ収集 |
| おおくぼ眼科 | 院長 | 大久保彰※ | データ収集 |
| 斎藤眼科医院 | 院長 | 斎藤信一郎※ | データ収集 |
| たかはし眼科 | 院長 | 高橋雄二※ | データ収集 |
| JCHO東京新宿メディカルセンター | 眼科/部長 | 間山千尋 | データ収集 |
| 横浜南共済病院 | 眼科/医長 | 井田泰嗣 | データ収集 |
| 大宮七里眼科 | 院長 | 山崎健一朗※ | データ収集 |
| きくな湯田眼科 |  | 湯田健太郎※ | データ収集 |
| ハートライフ病院 | 眼科/部長 | 親川格 | データ収集 |
| 金沢大学 | 眼科/病院臨床准教授 | 小林顕 | データ収集 |
| 大宮はまだ眼科 | 院長 | 濱田直紀※ | データ収集 |
| 高田コンタクトクリニック | 院長 | 本山祐大 | データ収集 |
| 羽生田眼科医院 | 院長 | 羽生田直人 | データ収集 |
| 原眼科医院 | 院長 | 原正※ | データ収集 |
| とつか眼科 | 院長 | 藤岡伸欣※ | データ収集 |
| お花茶屋眼科 |  | 柳靖雄 | データ収集 |
| 横浜市立大学附属市民総合医療センター | 眼科/客員教授 | 柳靖雄 | データ収集 |
| 三楽病院 | 眼科/眼科部長 | 中村真一 | データ収集 |
| 日本大学病院 | 眼科/准教授 | 田中公二 | データ収集 |
| 日本大学医学部附属板橋病院 | 眼科/准教授 | 林孝彦 | データ収集 |
| 名古屋大学 | 眼科/講師 | 上野真治 | データ収集 |
| 大阪大学 | 眼科/特任教授 | 川崎 良 | データ収集 |
| The University of Brescia | 眼科/准教授 | Vito Romano | データ収集 |
| 鹿児島大学 | 眼科/講師 | 園田祥三 | データ収集・解析 |
| 三重大学 | 眼科/助教 | 一尾享 | データ収集・解析 |
| 山梨大学 | 眼科/准教授 | 柏木賢治 | データ収集・解析 |
| National University of Singapore | 眼科/准教授 | 柳靖雄 | データ収集・解析 |
| ツカザキ病院 | 眼科/主任部長 | 田淵仁志 | データ収集・解析 |
| 弘前大学 | 教授 | 上野真治 | データ収集・解析 |
| 国立情報学研究所 | 所長 | 喜連川優 | データ解析 |
| The University of Tennessee | 眼科/講師 | Siamak Yousefi | データ解析 |
| Stefan cel Mare University of Suceava | コンピュータ、電子、自動化部門/専門員 | Alexandru Lavric | データ解析 |
| Federal University of Sao Paulo | 眼科視科学科/准教授 | Rosen M. Hazarbassanov | データ収集・解析 |
| University of Baghdad | 医用生体工学/講師 | Ali H.Al-Timemy | データ解析 |
| Universiti Kebangsaan Malaysia | Faculty of Information Science and Technology /上級講師 | Zaid Alyasseri | データ解析 |
| 株式会社NitroSquare | 代表取締役 | 島田圭 | ソフトウェア製作 |
| DeepEyeVison株式会社 | 代表取締役 | 髙橋秀徳 | 実用化 |

3. 研究の種類等

（1）研究の種類

■臨床研究

　■特定臨床研究に該当しない

□ヒトゲノム・遺伝子解析研究（生殖細胞系列変異又は多型を対象とするもの）

□上記以外の研究

（2）介入・侵襲の有無

介入：□あり　　　　　　　　　　　■なし

侵襲：□あり（軽微な侵襲を除く）　■なし（軽微な侵襲を含む）

4. 研究の背景及び意義

・機械学習による診断補助・治療方針決定補助の開発が盛んだが、一部の地域のみのデータで学習すると、その地域では有用な補助が出来ても、その他の地域でも有用とは限らない。そこでなるべく幅広い地域でデータを収集し、どの地域でも有用になるように開発することが望ましい。

5. 研究目的

・多施設の医用画像をまとめてビッグデータとして機械学習を行い、新規の診断補助・治療方針決定補助を開発する。

6. 研究デザイン等

（1）　 研究デザイン

　　　　・研究の種類：後ろ向き研究

　　　　・研究手法：データベース利用

　　　　・多機関・国際共同研究

（2）　 科学的合理性の根拠

・意義：疾患には人種差があるため、なるべく幅広い地域からデータを収集する事でどの地域でも有用な診断補助・治療方針決定補助をする人工知能を開発できると考えられる。

・統計解析の方法：機械学習アルゴリズムと同じ画像をヒトも判定し比較するため、対応のある検定となる。

・評価の項目：機械学習の性能は正答率で評価される。

・方法：窃盗・ハッキングの対策を鍵と暗号化で十分した上で、万が一の漏洩を考え収集する個人情報を必要最小限にし、出力は更に個人情報を減らした。

7. 評価項目

・Primary Outcome：機械学習による推論の正答率

・Secondary Outcome：なし

8. 統計解析の方法

・ヒトと機械学習の間で対応のあるt検定

9. 目標症例数及び設定根拠

目標症例数：研究全体で523,000人（そのうち自治医大150,000例）

設定根拠：深層学習はビッグデータが必要であり、例えばGoogleの発表した糖尿病網膜症病期判定論文では学習する画像を増やすと5万枚程度までは診断率が上がることが示されている。医用画像には眼科画像だけでもカラー眼底写真の他に蛍光眼底写真・自発蛍光眼底写真・広角眼底写真・光干渉断層計像・前眼部写真・蛍光前眼部写真・超音波Bモード像・前眼部光干渉断層計像等があり、日常診療では全ての撮影をすることはないため10倍の52万3000人に設定した。

10. 対象集団

（1）　 対象者

①　人数、種別

■患者（全体で約423,000人、そのうち本学の対象患者　約50,000人）

具体的疾患名等：網膜変性・視神経萎縮・白内障・前眼部炎症・眼付属器炎症と診断された患者全例

■自治医科大学附属病院、又は、さいたま医療センターの外来患者

■自治医科大学附属病院、又は、さいたま医療センターの入院患者

■上記以外の機関（機関名：青木眼科医院・おおくぼ眼科・斎藤眼科医院・たかはし眼科・JCHO東京新宿メディカルセンター・横浜南共済病院・鹿児島大学・三重大学・山梨大学・National University of Singapore・大宮七里眼科・きくな湯田眼科・ハートライフ病院・金沢大学・ツカザキ病院・大宮はまだ眼科・高田コンタクトクリニック・羽生田眼科医院・原眼科医院・とつか眼科・お花茶屋眼科・横浜市立大学附属市民総合医療センター・三楽病院・日本大学病院・日本大学医学部附属板橋病院・名古屋大学・Federal University of Sao Paulo・大阪大学・弘前大学・The University of Brescia）の外来患者

■上記以外の機関（機関名：青木眼科医院・おおくぼ眼科・斎藤眼科医院・たかはし眼科・JCHO東京新宿メディカルセンター・横浜南共済病院・鹿児島大学・三重大学・山梨大学・National University of Singapore・大宮七里眼科・きくな湯田眼科・ハートライフ病院・金沢大学・ツカザキ病院・大宮はまだ眼科・高田コンタクトクリニック・羽生田眼科医院・原眼科医院・とつか眼科・お花茶屋眼科・横浜市立大学附属市民総合医療センター・日本大学病院・日本大学医学部附属板橋病院・名古屋大学・Federal University of Sao Paulo・大阪大学・弘前大学・The University of Brescia）の入院患者

■その他（約100,000人、そのうち本学の対象者　約100,000人）

具体的に対象とする者： 本学健診センターを受診する健常者

②　対象年齢

■限定なし

□限定あり（　　　歳　～　　　歳）

③　性別

□男　　　□女　　　■両性

（2）　 適格基準

選択基準：眼科画像を撮影した患者と健診受診者

除外基準：除外を希望した患者と健診受診者

11. 研究期間

研究期間：臨床研究等許可決定後～2028年12月31日まで

登録期間：2002年01月01日～2027年12月31日まで

12. 研究方法

・本学眼科を含むデータ収集施設にて2002年1月1日から2027年12月31日までの間の日常診療で撮影した眼科画像・動画あるいは健康診断で撮影した医用画像を収集する。診療録から性別・年齢・視力・屈折・眼圧・所見・診断・治療内容を調査し、データベースを構築する。収集は随時行い、機械学習を都度行い、作成されたアルゴリズムまたは提供されたアルゴリズム（キヤノンの他国承認済みAI）の性能を解析する。本研究のようなデータベース作成研究の多くが、作成に要した公的研究費の終了と同時に活動停止して血税が無駄になっているとの批判があり、今回10年と区切り良く申請しているが望ましくはデータの保存形式や倫理基準をアップデートさせながら収集と機械学習を続け、データ収集地域の医療に貢献していくべきと考えている。

　　　　2002年1月1日　　　許可日　　　　　　　　　　　　　　2028年12月31日

撮影

データ収集 ↓↓↓↓↓↓↓↓↓↓↓↓↓↓↓↓↓↓↓↓↓

解析　　　　　　　 　　　　　∨

　　　　　　　　　　　　　　　　　　　　　　　∨

　　　　　　　　　　 　　　　　　　　　　　　　　　　　　　　　　　　∨

　　　　　　　　　　　　　　　　　　　　　　　　　　　　　　2027年12月31日

解析イメージ3通り。撮影は研究とは無関係な日常診療。データ収集は許可を得てから随時行うので「↓」で示した。許可日に行う解析は2002年から許可日までのデータ、2本目の「→」のみで行う。データ収集最終日に行う解析は2002年からその日までのデータ、4本目の「→」で行う。間の解析はその時点でデータベースに入っている情報、3本目の「→」で行う。

・データ収集施設は医用画像と情報を自治医科大学に提供する。自治医科大学は自施設のデータと提供された他施設のデータを自治医科大学眼科で管理するデータベースに保管する。

・データ解析施設は自治医科大学に必要なデータを希望し、自治医科大学は希望のあったデータをデータベースから出力させ電子的に提供し、データ解析施設はその提供された情報を用いて機械学習を行い、結果を解析し、臨床データでAIの効果・リスクを検証する。海外含めデータが送付されるのはデータが整う都度であり、データ解析施設に企業が含まれる場合は許可された日程を超えて解析されないように共同研究契約で日程を制限し、情報送付記録を残すことで、解析結果の追試・論文発表を可能とし、透明性を担保する。

データの流れ

データ収集のみ施設

データ収集・解析施設

自治医科大学

データ解析のみ施設

上記3種類の施設については、各施設で要する研究費用は各施設で負担する。将来知的財産・特許が発生した際は開発した施設の研究者が発明者となるが、その施設が受領したデータの前処理に、特許に必要な技術が含まれていた場合は、その処理を着想し行った、または着想してデータ収集施設に依頼した、自治医科大学の研究者も発明者となる。その際の按分・寄与率の算定は、開発した施設と自治医科大学の間で、各々発生した費用の割合を基本とし、協議のうえ合意により決定する。データ提供のみでは寄与した事にならない。

その他の施設①：「ソフトウェア製作」の株式会社NitroSquareは、データ解析に関わらず機械学習用のソフトウェア製作を行い、自治医科大学と共同で機械学習を行う。研究費用は自治医科大学眼科から株式会社NitroSquareに300万円を支払い、ソフトウェアを株式会社NitroSquareが製作する。前述の経費とは別に、株式会社NitroSquareが300万円自己負担することが見込まれているが、詳細は自治医科大学眼科と株式会社NitroSquareが協議の上合意により定められる。共同研究の結果生じた発明等に係る知的財産権は自治医科大学所有とする。

その他の施設②：「実用化」のDeepEyeVision株式会社は、データ解析に関わらず研究機器の整備を行い、自治医科大学と共同で機械学習を行い、特許第6745496号その他のDeepEyeVision株式会社が保有する特許権や本研究で期待される将来の特許を核とする技術(ノウハウ・アルゴリズム)の実用化を行う。共同研究の結果生じた発明等（DeepEyeVision株式会社が保有する特許等の知的財産権を除く。）が実用化された後は技術ライセンス料を自治医科大学に支払う。共同研究の結果生じた発明等に係る知的財産権は共有とし、その持分比は、当該発明等に対する貢献度に応じて、協議のうえ合意により決定する。

なおキヤノン株式会社は他国承認済みAI(EyeArt®：別紙参照)の試用目的提供のみ行い研究を行わない。

・自前のコンピュータよりクラウドの方が機械学習スピードとコストが優位であり、クラウドに必要データのみアップロードして解析する（委託と表現しているが、委託業者はクラウドを提供するのみで機械学習自体は各データ解析施設が行う）。クラウド業者は委託業者の項目に記載の認証を取得しており、自治医科大学内のストレージにデータを保管するのと同等以上の安全性が担保されている (委託と表現しているが、委託業者はクラウドストレージを提供するのみで保管作業や漏洩を防止するための設定や暗号化は自治医科大学眼科が行う)。

・米国のHIPAA法（米国における医療保険の相互運用性と説明責任に関する法令：厚生労働省内の審議に使われた文書添付）における個人情報等の保護に関する規定に準拠する。

・研究代表者は人工知能学会会員であり、自身だけでなく研究全体で人工知能学会倫理指針（添付）を遵守する。

13. インフォームド・コンセントを受ける手続等

（1）　 試料・情報等の収集について

□新たな試料・情報等を使用する

　　　 その内容：

　　　　 □文書で同意を得る

□口頭＋記録で同意を得る

　その理由と対応策：

□情報公開＋オプトアウトで拒否できる権利を保障する

　　　　　　 　その理由と対応策：

　　　　　 □その他

　　　　　　 　その理由と対応策：

■既存試料・情報等を使用する

　その内容：眼科画像・動画・性別・年齢・視力・屈折・眼圧・所見・診断・治療内容・以下本学健診センターのみ、胸部X線・マンモグラフィー・CT（頭部・胸部・腹部）・EGD（上部消化管内視鏡）・腹部超音波・乳腺超音波・MDL（上部消化管バリウム造影）・ECG（心電図）

□人体から取得された試料を用いる

□文書で同意を得る

□口頭＋記録で同意を得る

□情報公開＋オプトアウトで拒否できる権利を保障する

その理由と対応策：

■情報を用いる

□文書で同意を得る

□口頭で同意を得る

■オプトアウトで拒否できる権利を保障する

その理由と対応策：指針「第４章　第８インフォームド・コンセントを受ける手続等１（２）イ（ウ）、および（５）ウに該当する情報であるので、情報公開(添付)・オプトアウトで同意を得たこととし、病院長等に提供の報告を行う。本研究が承認された後、眼科ホームページとさいたま医療センターホームページ、健診センターホームページ、各共同研究機関のホームページ上に情報公開文書を掲載する。

□口頭で説明はせず、説明文書も渡さない

その理由と対応策：

（2）　 代諾者等の要件等

□代諾者等をおく

＜代諾者等の選定方針＞

①　研究対象者の種類及び理由

（ア）種類

　　　　　　　　□未成年（□16歳未満　□中学校等の課程を修了している者又は16歳以上）

□インフォームド・コンセントを与える能力を欠くと客観的に判断される成人

□死者

□その他（　　　　　　　　　　　　　　　　　　　　　　　　　　　　　　）

（イ）当該者を研究対象者とすることが必要な理由：

②　代諾者等の種類

□親権者　　□配偶者　　□父母 □成人の子

□その他（　　　　　　　　　　　　　　　　　　　）

③　代諾者等への説明事項

□研究対象者等への説明と同一事項

□研究対象者等への説明とは別事項

　　　　　　　　具体的な説明事項：

■代諾者等をおかない

　　　　　＜その理由＞

■代諾者等をおく研究ではない

　　　　　□代諾者等をおく研究であるが、以下の全てに該当することから、本人の同意を得るため。

□研究対象者は未成年である。ただし、中学校等の課程を修了している、又は16歳以上未成年者であり、かつ、研究を実施されることに関する十分な判断能力を有すると判断される

□侵襲を伴わない研究

□研究の目的及び試料・情報の取扱いを含む研究の実施についての情報を公開し、本研究が実施又は継続されることについて、研究対象者の親権者又は未成年後見人が拒否できる機会を保障している

　　　　　　　　具体的な対応：

（3）　 インフォームド・アセントを得る手続き

□インフォームド・アセントを得る

説明事項及び方法：

■インフォームド・アセントを得ない

　　　　　■インフォームド・アセントを得る必要がない

□インフォームド・アセントを得る必要があるが、以下の理由により得ない

理由：

14. 有害事象及び重篤な有害事象

（1）　 有害事象及び重篤な有害事象の定義

・なし

（2）　 重篤な有害事象が発生した場合の対応

　・有害事象はない

15. 倫理的・要配慮事項等

15.1 遵守すべき規制等

・本研究は、ヘルシンキ宣言に基づく倫理的原則に則り、人を対象とする生命科学・医学系研究に関する倫理指針を遵守して実施する。

15.2 個人情報等の取扱い

（1）　 収集する試料・情報等について

□試料（血液・ヒト組織等）を収集する

■試料は収集しない

■情報を収集する

　　　　　　　■診療情報

　　　　　　　□質問紙等

　　　　　　　□その他（　　　　　　　　　　）

□情報は収集しない

（2）　 試料・情報等の匿名化

■試料・情報等を匿名化する

□匿名化（対応表なし）

　　　 　　匿名化の時期：　□研究開始時　　□研究期間中の一定の時期　　□研究終了時

　　　　 　匿名化の方法：

■匿名化（対応表あり）

　　　　 　匿名化の時期：　■研究開始時　　□研究期間中の一定の時期　　□研究終了時

　　　　 　匿名化の方法：1日違うだけで視力1.2から失明にまで変わりうるから診療日をデータベースに入力する必要はあるが、正確な受診日付は個人を特定しうるので各患者の最初に入力したデータの測定日を各月の16日に変更し、他のデータの日付もそれに合わせて変更して出力する事で、データの季節は分かっても何日に受診したかは分からなくなる。年齢は診断・治療に重要であり、データベースは撮影時の年齢を出力する必要があるが、生年月日は個人を特定しうるので入力はしても出力はしない。撮影時の年齢のみ出力する。新生児期は生まれてからの日数が重要であるから生後何日かを出力する。撮影日の出力は変更されている事から生年月日は特定されない。虹彩結節や虹彩萎縮など虹彩所見は診断に重要である。虹彩紋理画像は個人認証を目的に特徴抽出を行えば個人情報となるが、診療目的の既存データを用いるので個人識別に使用できるような虹彩全体が明瞭に写った写真はない。また、健診センターは虹彩を撮影しない。万が一あれば一部マスクして識別に使用できないようにする。各施設の患者IDや撮影日が含まれている事の多い眼底写真ファイル名は、暗号化して加工する。写真自体に日付などが映り込んでいた場合はマスクする（ソフト開発済み）。実際に解析するデータセットには患者を特定しうる個人情報は残さず、眼科画像・動画・性別・年齢・視力・屈折・眼圧・所見・診断・治療内容・以下本学健診センターのみ、胸部X線・マンモグラフィー・CT（頭部・胸部・腹部）・EGD（上部消化管内視鏡）・腹部超音波・乳腺超音波・MDL（上部消化管バリウム造影）・ECG（心電図）のみをデータベースは出力する。患者ID匿名化の対応表は暗号化し、各施設の研究責任者が各施設各部門において鍵の掛かるキャビネットに保管するパスワードを用いた時のみ復号されるようにすることで、各施設が自施設の患者のみIDが分かるようにし、各施設は本学へデータを提供するが、対応表等は提出しない。

対応表を作成する理由

■匿名後に研究の正確性、科学性及び質を担保するために必要時にデータの修正・変更又は追加を行う可能性があり、追跡可能とする

■対象者より同意撤回や参加拒否申し出時、該当データを同定する必要があるため

□その他(具体的に記載して下さい)

理由：

□試料・情報等を匿名化しない

試料・情報等の種類：

　 匿名化しない理由：

（3）　 試料・情報等の共同利用

□共同研究機関との試料・情報の授受がない

■共同研究機関との試料・情報等の授受がある

■国内の研究機関　　■（提供先の機関：鹿児島大学・三重大学・山梨大学・国立情報学研究所・株式会社NitroSquare・DeepEyeVision株式会社・ツカザキ病院・弘前大学）

（当該提供に係る責任者：園田祥三・一尾享・柏木賢治・喜連川優・今泉英明・島田圭・髙橋秀徳・田淵仁志・上野真治）

■（提供元の機関：自治医科大学附属さいたま医療センター・青木眼科医院・おおくぼ眼科・斎藤眼科医院・たかはし眼科・JCHO東京新宿メディカルセンター・横浜南共済病院・鹿児島大学・三重大学・山梨大学・大宮七里眼科・きくな湯田眼科・ハートライフ病院・金沢大学・ツカザキ病院・大宮はまだ眼科・高田コンタクトクリニック・羽生田眼科医院・原眼科医院・とつか眼科・お花茶屋眼科・横浜市立大学附属市民総合医療センター・三楽病院・日本大学病院・日本大学医学部附属板橋病院・名古屋大学・大阪大学・弘前大学）

（当該提供に係る責任者：蕪城　俊克・青木真祐・大久保彰・斎藤信一郎・高橋雄二・間山千尋・井田泰嗣・園田祥三・一尾享・柏木賢治・山崎健一朗・湯田健太郎・親川格・小林顕・田淵仁志・濱田直紀・本山祐大・羽生田直人・原正・藤岡伸欣・柳靖雄・柳靖雄・中村真一・田中公二・林孝彦・上野真治・川崎良・上野真治）

項目：眼科画像・動画・性別・年齢・視力・屈折・眼圧・所見・診断・治療内容・以下本学健診センターのみ、胸部X線・マンモグラフィー・CT（頭部・胸部・腹部）・EGD（上部消化管内視鏡）・腹部超音波・乳腺超音波・MDL（上部消化管バリウム造影）・ECG（心電図）

試料・情報等について他機関との授受が行われる場合の手続き：

本学において研究実施許可を受けた後、本学で一括審査を受けた共同研究機関以外の機関については、共同研究者から共同研究機関の長に、許可を受けた本学の研究計画書を提出し、本学の研究計画書にそって当該共同研究機関において研究を実施する許可を文書（当該機関の長から当該共同研究者に対する研究実施許可書）で受ける。データベース出力情報（表形式と画像ファイル。対応表等は含まれない）を暗号化してクラウド(委託業者に列挙)経由で、サイズが小さい場合はメール添付で、送付する。授受の記録を残す。

・対応表等の管理方法と具体的な対応：

患者IDの対応表等は暗号化し、各施設の研究責任者が各施設各部門において鍵の掛かるキャビネットに保管するパスワードを用いた時のみ復号されるようにすることで、各施設が自施設の患者のみIDが分かるようにし、各収集施設は本学へ、本学は各解析施設へデータを提供するが、対応表等は提出しない。

■海外の研究機関　　■（提供先の機関：National University of Singapore・The University of Tennessee・Stefan cel Mare University of Suceava・Federal University of Sao Paulo・University of Baghdad・Universiti Kebangsaan Malaysia）

　　　　　　　　　　　　　　　　　（当該提供に係る責任者：柳靖雄・Siamak Yousefi・Alexandru Lavric・Rosen M. Hazarbassanov・Ali H.Al-Timemy・Zaid Alyasseri）

　■（提供元の機関：National University of Singapore・Federal University of Sao Paulo・The University of Brescia）

　　　　　　　　　　　　　　　　　（当該提供に係る責任者：柳靖雄・Rosen M. Hazarbassanov・Vito Romano）

項目：眼科画像・動画・性別・年齢・視力・屈折・眼圧・所見・診断・治療内容・以下本学健診センターのみ、胸部X線・マンモグラフィー・CT（頭部・胸部・腹部）・EGD（上部消化管内視鏡）・腹部超音波・乳腺超音波・MDL（上部消化管バリウム造影）・ECG（心電図）

試料・情報等について他機関との授受が行われる場合の手続き：

データベース出力情報（表形式と画像ファイル。対応表等は含まれない）を暗号化してクラウド(委託業者に列挙)経由で、サイズが小さい場合はメール添付で、送付する。授受の記録を残す。

■共同利用する試料・情報等に個人情報を含む

その内容：性別・年齢

個人情報を利用する理由：性別と年齢は日常診療でも診断・治療方針決定に使用する情報であり、人工知能も使用した方がより正確に診断補助・治療方針決定補助できると考えられるため。

15.3 予測されるリスク、利益及びリスクを最小化する対策等

（1）　 研究対象者に生じる負担及び予測されるリスク

①　費用負担

□研究に参加することにより、研究対象者に追加の費用負担がある

□全額自己負担（負担額：約　　　　　円）

□一部自己負担（負担額：約　　　　　円）

□保険診療の範囲内

□その他（　　　　　　　　　　　　　　）

■研究に参加した場合、研究対象者に追加の費用負担はない

②　その他の負担及び予測されるリスク

□研究に参加した場合、研究対象者にその他の負担がある

　　　　　　　具体的事項：

■研究に参加した場合、研究対象者にその他の負担はない

（2）　 研究対象者に生じる利益

①　対象者への謝礼

　　　　　　□あり（具体的に：　 　　　　　　　　　　　　　　　）

　　　　　　■なし

②　その他の利益

　　　　　　□あり　内容：

　　　　　　■なし

（3）　 研究対象者に生じる負担並びに予測されるリスク及び利益の総合的評価、並びに当該負担及びリスクを最小化する対策

①　総合的評価

　・リスクも利益もない。

②　負担及びリスクを最小化する対策

　・なし。

③　損失補償

□本研究の実施によって発生する損失に対する補償がある

補償の内容：

■本研究の実施によって発生する損失に対する補償はない

15.4 研究対象者に係る研究結果の取扱い

■研究対象者の健康、子孫に受け継がれ得る遺伝的特徴等に関する重要な知見が得られる可能性はない

□研究対象者の健康、子孫に受け継がれ得る遺伝的特徴等に関する重要な知見が得られる可能性がある

研究結果の取扱い：

15.5 学長への報告内容及び方法（重篤な有害事象の報告は除く）

■毎年1回、臨床研究等の進捗状況並びに有害事象及び不具合等の発生状況を臨床研究等進捗状況報告書により遅滞なく学長あてに報告する^※^

■臨床研究等が終了（中止）したときは、速やかに臨床研究等終了報告書により学長あてに報告する^※^

■臨床研究が終了（中止）した時に、試料・情報を他機関に提供した場合には、他の研究機関への試料・情報の提供に関する届出書により遅滞なく学長等に報告する

■臨床研究が終了（中止）した時に、他機関から試料・情報の提供を受けた場合には、他の研究機関からの試料・情報の受領に関する届出書により遅滞なく学長等に報告する

■試料・情報の授受に関する記録を廃棄した場合には、速やかに試料・情報等廃棄報告書により学長に報告する

15.6 研究の資金源等、研究に係る利益相反及び個人の収益等、研究者等の研究に係る利益相反に関する状況

（1）　 資金源

　　　 ■講座研究費

　　　 □受託研究費 依頼者：

■公的研究費 補助金名：基盤C一般21K09751「眼底画像とサイトカイン濃度のAI解析による加齢黄斑変性病態の解明基盤構築(2021-2023)・若手21K16903「深層学習を用いた視野検査結果予測」(2021-2022)

　　　 ■その他：共同研究費

（2）　研究者等の関連組織との関わり

　・記載した共同研究機関と研究業務委託先以外に関連組織はない。

　・株式会社NitroSquareと研究者との間には個人的な関係（親族関係や顧問など）はない。同社はデータ解析には関わらずソフトウェア製作と機械学習を行う。

・研究分担者の髙橋秀徳はDeepEyeVision株式会社の創業株主で代表取締役である。同社はデータ解析には関わらず研究機器整備と機械学習を行う。また同社は本研究で期待される将来の特許を核とする技術(ノウハウ・アルゴリズム)の使用あるいは買い取りを検討しており、特許を核とする技術の実用化に関わる。

・キヤノン株式会社と研究者との間には個人的な関係（親族関係や顧問など）はない。同社は他国承認済みAI(EyeArt®：別紙参照)の試用目的提供のみ行うため共同研究契約はない。本AIは本研究のために特別に提供されるものではなく、他の研究機関にも提供されうる。担当者は事業企画担当木倉龍太。

（3）　 利益相反の状況

　・自治医科大学眼科学講座・株式会社NitroSquareの二者共同研究においては各々年300万円を負担し、機械学習を行う。自治医科大学眼科学講座の300万円は株式会社NitroSquareに支払われる。

・自治医科大学眼科学講座・DeepEyeVision株式会社の二者共同研究においてはラベル付けの人件費の一部がDeepEyeVision株式会社から自治医科大学へ支払われる、あるいはラベル付け要員が派遣される。本研究で期待される将来の特許を核とする技術(ノウハウ・アルゴリズム)の使用あるいは買い取りの際は、DeepEyeVision株式会社から自治医科大学に使用料あるいは対価が支払われる。

（4）　 研究によって得られる利益

■あり　その経済的利益の拠出機関（企業名等）：DeepEyeVision株式会社

その経済的利益の帰属先：□研究者個人　■講座名等（眼科学）

■その他（自治医科大学）

□なし

（5）　 特許権等

■特許権等が発生する可能性がある

　　　　　その特許権等の帰属先：■自治医科大学　　■研究者個人

　　　　　　　　　　　　　　　　■その他（各共同研究機関）

□特許権等が発生する可能性はない

15.7 研究に関する情報公開の方法

（1）　 研究概要及び結果の登録

□研究の概要及び結果を登録する

　　　　　登録先　　□厚生労働省が整備するデータベース（jRCT）

□国立大学附属病院長会議（UMIN）

　　 　　□一般社団法人日本医療情報センター

□公益財団法人日本医師会

■研究の概要及び結果を登録しない

具体的理由：観察研究であるから。

（2）　 研究で新たに得られる個人データ（検査結果等）の開示

■本研究で新たに得られる個人データはない

□本研究で新たに得られる個人データがある

①　研究対象者への開示

□研究対象者に開示する

□原則として開示　　□希望者に開示

□研究対象者に開示しない

開示しない理由：

②　代諾者への開示

□代諾者に開示する

□原則として開示　□研究対象者の同意を条件として開示　□希望者に開示

□代諾者に開示しない

開示しない理由：

□本研究では代諾者はいない

③　家族等への開示

□家族等（遺族を含む）に開示する

□原則として開示　□研究対象者の同意を条件として開示　□希望者に開示

□家族等（遺族を含む）に開示しない

開示しない理由：

（3）　 研究成果の公開

■研究成果を公開する

　　　　　　公開の方法：■論文発表　　■学会発表　　■インターネット掲載

□その他（　　　　　　　　　　　　　　　　　　　　）

□研究成果は公開しない

　　　　　理由：

15.8 研究対象者に緊急かつ明白な生命の危機が生じている状況における研究の取扱い

■研究対象者に緊急かつ明白な生命の危機が生じている状況における研究ではない

□研究対象者に緊急かつ明白な生命の危機が生じている状況における研究である

□研究対象者に緊急かつ明白な生命の危機が生じている

□介入を行う研究であり、通常の診療では十分な効果が期待できず、研究の実施により研究対象者の生命の危機が回避できる可能性が十分にあると認められる

□研究の実施に伴って研究対象者に生じる負担及びリスクが必要最小限のものである

□代諾者又は代諾者となるべき者と直ちに連絡を取ることができない

15.9 健康被害に対する補償の有無及びその内容

■侵襲（侵襲がない、又は軽微な侵襲がある）を伴わない研究 [補償の対象外の研究]

□侵襲（軽微な侵襲を除く）を伴う研究 [補償の対象となる研究]

□補償あり

具体的内容：

□補償なし

理由：

15.10研究実施後における医療の提供に関する対応

■通常の診療を超えないあるいは医療行為を伴わない研究

□通常の診療を超える医療行為を伴う研究

研究実施後の医療の提供について具体的方法：

16. 試料・情報等の保管及び廃棄

（1）　 研究中の試料・情報等及び試料・情報の保管

　　①　試料・情報等の種類

■原試料・原資料（症例報告書、調査票等）

■加工した資料

□同意書

■匿名化対応表

■その他（暗号鍵）

　　②　試料・情報等の形態

□紙媒体の情報

■電子化した情報

□試料

□その他（　　　　　　　　　　）

　　③　保管場所

　　　　　■自治医科大学（具体的な場所：眼科学講座医局キャビネット　■施錠可能　□施錠不可能）

　　　　　■共同研究機関（具体的な場所：2.3で定義される共同研究各施設で定められたキャビネット　■施錠可能　□施錠不可能）

　　　　　□その他（具体的な場所：　　　　　　　　　　　　　　□施錠可能　□施錠不可能）

（2）　 研究終了後の試料・情報等の保管

□研究終了後、本申請の研究目的以外の目的に使用するため試料・情報等を保管する

　　　　　試料・情報等の種類：

　　　　　保管が必要な理由：

□保管した試料・情報等を別の目的に使用する際は、改めて倫理審査委員会に申請し承認を得る

□保管した試料・情報等を別の目的に使用する際に、改めて研究対象者の同意を得る

□保管した試料・情報等を別の目的に使用する際に、改めて研究対象者の同意を得ない

　　　改めて同意を得ない理由：

　　　　　保管場所

　　　　　　□自治医科大学（具体的な場所：　　　　　　　　　　□施錠可能　□施錠不可能）

　　　　　　□共同研究機関（具体的な場所：　　　　　　　　　　□施錠可能　□施錠不可能）

　　　　　　□その他（具体的な場所：　　　　　　　　　　　　　□施錠可能　□施錠不可能）

　　□研究終了後、一定期間（　　　　　か月）保存した後に破棄・廃棄する

　　試料・情報等の種類：

　　　　　保管場所

　　　　　　□自治医科大学（具体的な場所：　　　　　　　　　　□施錠可能　□施錠不可能）

　　　　　　□共同研究機関（具体的な場所：　　　　　　　　　　□施錠可能　□施錠不可能）

　　　　　　□その他（具体的な場所：　　　　　　　　　　　　　□施錠可能　□施錠不可能）

□侵襲（軽微な侵襲を除く）を伴い介入を行う研究であるため、研究の終了について報告された日から5年を経過する日、又は当該研究の結果の最終公表について報告された日から3年を経過した日のいずれか遅い日までの期間保管し、当該期間経過後は破棄・廃棄する

■研究終了後、直ちに試料・情報等を破棄・廃棄する

（3）　 試料・情報等の破棄・廃棄の方法

□オートクレーブ滅菌後焼却

□シュレッダーにて細断

■その他（データ消去専用ソフトウェアでストレージからデータを消去して廃棄する）

（4）　 試料・情報等の授受に関する記録の保管

□他機関との試料・情報の授受なし

■他機関との試料・情報の授受あり

　　　　　　■他機関への試料・情報の授受を行う研究であるため、当該試料・情報の提供をした日から3年を経過した日まで試料・情報の授受の記録について保管する

■他機関から試料・情報の提供を受けて研究を実施しようとする研究であるため、研究終了について報告された日から5年を経過した日まで試料・情報の授受の記録について保管する

（5）　 試料・情報等の保管及び廃棄の方法に関する報告書

■研究終了後、試料・情報等を保管している場合は試料・情報等保管状況報告書により学長あてに報告する

■研究終了後の試料・情報等の保管方法について変更が生じた場合は、試料・情報等保管状況変更報告書により学長に報告する

■試料・情報等を廃棄した場合は、試料・情報等廃棄報告書により学長に報告する

17. モニタリング及び監査の体制並びに実施手順

17.1 モニタリング

　　　　■モニタリングを実施しない

　　　　□モニタリングを実施する

　　　　実施体制及び実施手順：

17.2 監査

　　　　■監査を実施しない

　　　　□監査を実施する

　　　　監査を実施する理由：

　　　　実施体制及び実施手順：

18. 研究に関する業務の委託状況

　　□研究に関する業務を委託しない

■研究に関する業務を委託する

　　　　■契約あり(予定)　　□契約なし

　　　　委託業務の内容：機械学習・データ保管

　　　　委託先の監督方法：誰のものかを分からないように暗号化により匿名化して対応表（暗号鍵）は提供しない。暗号が破られていないか定期的に確認する。遵守されていなければ対策が完了するまで引き上げる。以下に示す認証を取得しており秘密保持・個人情報保護が担保されているため別途秘密保持契約は不要となっている。

・ISO27001：情報セキュリティの国際規格。評価対象：情報セキュリティの基本方針、組織マネジメント、情報管理指標、物理保護、通信保護、アクセス制御 等。更新頻度：毎年

保管対象：眼科画像・動画・性別・年齢・視力・屈折・眼圧・所見・治療内容・以下本学健診センターのみ、胸部X線・マンモグラフィー・CT（頭部・胸部・腹部）・EGD（上部消化管内視鏡）・腹部超音波・乳腺超音波・MDL（上部消化管バリウム造影）・ECG（心電図）

保管方法：暗号化データ

責任者・保管場所：エクストリーム-D株式会社創業者・代表取締役・最高経営責任者柴田直樹（〒140-0002東京都品川区東品川２丁目２−２５ サンウッド品川天王洲タワー205）、Amazon Web Services, Inc.（https://aws.amazon.com）、Microsoft Azure（https://azure.microsoft.com）、google（https://www.google.co.jp）、Dropbox (https://dropbox.com)、slack (https://slack.com)、box (https://box.com)

19. 研究対象者等及びその関係者が研究に係る相談を行うことができる体制及び相談窓口

（1）　 遺伝カウンセリングの必要性及びその体制

　・なし

（2）　 問い合わせ先

所属：眼科

職名：教授　氏名　川島秀俊

電話番号：0285-58-7382

学内内線番号：3526　PHS（所有している場合）：7503

e-mail：hidemeak＠jichi.ac.jp

（3）　 苦情申出先

自治医科大学附属病院臨床研究センター管理部（電話：0285-58-8933）
