# Supplementary material for: Predicting central choroidal thickness from colour fundus photographs using deep learning
Source: PLoS One. 2024 Mar 29;19(3):e0301467. doi: 10.1371/journal.pone.0301467 (PMC10980193; doi:10.1371/journal.pone.0301467)
Supplement: S2 File — (DOCX) [file pone.0301467.s002.docx]

**Clinical research plan**

1. Research topic name　　　Building a database for machine learning of medical images

2. Research implementation system

2.1 Overview of implementation system

□Jichi Medical University independent research

■ Multi-institutional joint research led by Jichi Medical University

2.2 Implementation structure within the university

|  | Affiliation | job title | full name | Roles and responsibilities |
| --- | --- | --- | --- | --- |
| ①Research director | ophthalmology | Professor | Hidetoshi Kawashima | General management/data management |
| ② Researcher | ophthalmology | Associate Professor | Hidenori Takahashi | Machine learning (not involved in analysis or data management) |
|  | Health checkup center | Professor | Hiroshi Miyashita | Health checkup center data management |
|  | ophthalmology | Teacher | Yusuke Arai | data analysis |
|  | ophthalmology | Assistant professor | Meri Watanabe | data analysis |
|  | ophthalmology | Assistant professor | Shinichi Sakamoto | data analysis |
|  | ophthalmology | Assistant professor | Satoru Inoda | machine learning |
|  | ophthalmology | Hospital assistant professor | Yuka Kasuya | Data collection |
|  | ophthalmology | Hospital assistant professor | Kosuke Nagaoka | Data collection |
|  | ophthalmology | Clinical assistant professor | Yuto Hashimoto | Data collection |
|  | ophthalmology | Clinical assistant professor | Hana Yoshida | Data collection |
|  | ophthalmology | Clinical assistant professor | Takuya Takayama | Data collection |
|  | ophthalmology | photographer | Hironobu  Tampo | Data collection |
| ③Research collaborators |  |  |  |  |
|  |  |  |  |  |

<Attendance of education/training>

- Research director's attendance status, etc.

Ethics seminar: 2 July 09, 021

e-learning :

CREDITS [Ethics and Code of Conduct Course] 2 April 09 , 021

CREDITS [Clinical Research Implementation Course ] 2 April 09 , 021

■The research director confirmed that the co-researchers and collaborators attended the ethics seminar and e-learning .

2.3 Implementation structure of joint research institutions

| Institution name | Affiliation/Job title | full name | Roles and responsibilities |
| --- | --- | --- | --- |
| Saitama Medical Center Ophthalmology | Professor | Toshikatsu Bujo | Data collection |
| Aoki Eye Clinic | Director | Mayu Aoki* | Data collection |
| Okubo Ophthalmology | Director | Akira Okubo* | Data collection |
| Saito Eye Clinic | Director | Shinichiro Saito* | Data collection |
| Takahashi ophthalmology | Director | Yuji Takahashi* | Data collection |
| JCHO Tokyo Shinjuku Medical Center | Ophthalmology/Director | Chihiro Mayama | Data collection |
| Yokohama Minami Kyosai Hospital | Ophthalmology/Medical Director | Yasushi Ida | Data collection |
| Omiya Shichiri Ophthalmology Clinic | Director | Kenichiro Yamazaki* | Data collection |
| Kikuna Yuda Eye Clinic |  | Kentaro Yuda* | Data collection |
| heart life hospital | Ophthalmology/Director | Itaru Oyakawa | Data collection |
| Kanazawa University | Ophthalmology/Hospital Clinical Associate Professor | Akira Kobayashi | Data collection |
| Omiya is still an ophthalmologist | Director | Naoki Hamada* | Data collection |
| Takada Contact Clinic | Director | Yudai Motoyama | Data collection |
| Hanyuta Eye Clinic | Director | Naoto Hanyuta | Data collection |
| Hara Eye Clinic | Director | Original Masaru※ | Data collection |
| Totsuka Ophthalmology | Director | Nobuyoshi Fujioka* | Data collection |
| Ohanajaya Ophthalmology |  | Yasuo Yanagi | Data collection |
| Yokohama City University Citizens General Medical Center | Ophthalmology/Visiting Professor | Yasuo Yanagi | Data collection |
| Sanraku Hospital | Ophthalmology/Ophthalmology Department Director | Shinichi Nakamura | Data collection |
| Nihon University Hospital | Ophthalmology/Associate Professor | Koji Tanaka | Data collection |
| Nihon University School of Medicine Itabashi Hospital | Ophthalmology/Associate Professor | Takahiko Hayashi | Data collection |
| Nagoya University | Ophthalmology/Lecturer | Shinji Ueno | Data collection |
| Osaka University | Ophthalmology/Specially Appointed Professor | Ryo Kawasaki | Data collection |
| The University of Brescia | Ophthalmology/Associate Professor | Vito Romano | Data collection |
| Kagoshima University | Ophthalmology/Lecturer | Shozo Sonoda | Data collection/analysis |
| Mie University | Ophthalmology/Assistant Professor | Toru Ichio | Data collection/analysis |
| University of Yamanashi | Ophthalmology/Associate Professor | Kenji Kashiwagi | Data collection/analysis |
| National University of Singapore | Ophthalmology/Associate Professor | Yasuo Yanagi | Data collection/analysis |
| Tsukazaki Hospital | Ophthalmology/Chief Director | Hitoshi Tabuchi | Data collection/analysis |
| Hirosaki University | Professor | Shinji Ueno | Data collection/analysis |
| National Institute of Informatics | chief | Yu Kitsuregawa | data analysis |
| The University of Tennessee | Ophthalmology/Lecturer | Siamak Yousefi | data analysis |
| Stefan cel Mare University of Suceava | Computer, electronics, automation department/specialist | Alexandru Lavric | data analysis |
| Federal University of Sao Paulo | Department of Ophthalmology/Visual Science/Associate Professor | Rosen M. Hazarbassanov | Data collection/analysis |
| University of Baghdad | Medical bioengineering/Lecturer | Ali H.Al -Timemy | data analysis |
| University Kebangsaan Malaysia | Faculty of Information Science and Technology /Senior Lecturer | Zaid Alyasseri | data analysis |
| NitroSquare Co., Ltd. | CEO | Kei Shimada | software production |
| DeepEyeVison Co., Ltd. | CEO | Hidenori Takahashi | Practical realization |

3. Type of research, etc.

(1) Type of research

■Clinical research

■Not applicable to specific clinical research

□Human genome/gene analysis research (targeting germline mutations or polymorphisms)

□Research other than the above

(2) Presence or absence of intervention/invasiveness

Intervention: □Yes ■No

Invasion: □Yes (excluding minor invasion) ■No (including minor invasion)

4. Background and significance of the research

- Development of diagnostic aids and treatment policy decision aids using machine learning has been active, but if learning is done using data from only a certain region, even if the aid is useful in that region, it may not be useful in other regions. Therefore, it is desirable to collect data from as wide a region as possible and develop it in a way that will be useful in any region.

5. Research purpose

- Perform machine learning on medical images from multiple facilities as big data and develop new diagnostic aids and treatment policy decision aids.

6. Research design etc.

(1) Research design

・Research type: Retrospective study

・Research method: Database use

・Multi-institutional/international joint research

(2) Basis of scientific rationality

・Significance: Since there are racial differences in diseases, it is thought that by collecting data from as wide a range of regions as possible, it will be possible to develop artificial intelligence that will be useful in any region to assist in diagnosis and treatment policy decisions.

・Statistical analysis method: Humans also judge and compare the same images as the machine learning algorithm, so it is a paired test.

・Evaluation items: The performance of machine learning is evaluated by the correct answer rate.

・Method: After taking sufficient measures against theft and hacking using keys and encryption, we minimized the amount of personal information collected in case of leakage, and further reduced the personal information output.

7.Evaluation items

・Primary Outcome: Correct answer rate of inference by machine learning

・Secondary Outcome: None

8.Statistical analysis methods

・Paired t- test between humans and machine learning

9.Target number of cases and basis for setting

Target number of cases: 523,000 for the entire study (of which 150,000 at Jichi Medical University )

Setting rationale: Deep learning requires big data, and for example, a paper published by Google on diabetic retinopathy stage determination shows that increasing the number of images to be learned can improve the diagnosis rate up to about 50,000 images. Medical images include not only ophthalmological images but also color fundus photographs, fluorescent fundus photographs, autofluorescent fundus photographs, wide-angle fundus photographs, optical coherence tomography images, anterior segment photographs, fluorescent anterior segment photographs, ultrasound B-mode images, The number of patients was set at 523,000 patients, which is 10 times the number, because there are images of anterior segment optical coherence tomography, etc., and not all images are taken in daily medical treatment.

10.Target population

(1) Target audience

① Number of people, type

423,000 in total , of which approximately 50,000 are patients at our university )

Specific disease name, etc.: All patients diagnosed with retinal degeneration, optic nerve atrophy, cataract, anterior segment inflammation, and ocular adnexal inflammation.

Outpatients at Jichi Medical University Hospital or Saitama Medical Center

Inpatients at Jichi Medical University Hospital or Saitama Medical Center

Institutions other than the above ( institution names: Aoki Eye Clinic, Okubo Eye Clinic, Saito Eye Clinic, Takahashi Eye Clinic, JCHO Tokyo Shinjuku Medical Center, Yokohama Minami Kyosai Hospital, Kagoshima University, Mie University, Yamanashi University, National University of Singapore , Omiya Shichiri Eye Clinic , Kikuna Yuda Eye Clinic, Heart Life Hospital, Kanazawa University, Tsukazaki Hospital, Omiya Hamada Eye Clinic, Takada Contact Clinic, Hanyuta Eye Clinic, Hara Eye Clinic, Totsuka Eye Clinic, Ohanajaya Eye Clinic, Yokohama City University Citizens General Medical Center・Outpatients at Sanraku Hospital, Nihon University Hospital, Nihon University Itabashi Hospital, Nagoya University , Federal University of Sao Paulo , Osaka University, Hirosaki University, The University of Brescia )

Institutions other than the above ( institution names: Aoki Eye Clinic, Okubo Eye Clinic, Saito Eye Clinic, Takahashi Eye Clinic, JCHO Tokyo Shinjuku Medical Center, Yokohama Minami Kyosai Hospital, Kagoshima University, Mie University, Yamanashi University, National University of Singapore, Omiya Shichiri Eye Clinic, Kikuna Yuda Eye Clinic, Heart Life Hospital, Kanazawa University, Tsukazaki Hospital, Omiya Hamada Eye Clinic, Takada Contact Clinic, Hanyuta Eye Clinic, Hara Eye Clinic, Totsuka Eye Clinic, Ohanajaya Eye Clinic, Yokohama City University Citizens General Medical Center・Inpatients at Nihon University Hospital, Nihon University Itabashi Hospital, Nagoya University, Federal University of Sao Paulo , Osaka University, Hirosaki University, The University of Brescia )

■Others (approximately 100,000 people, of whom approximately 100,000 are from our university )

Specifically targeted people: Healthy people who visit the university's health checkup center

② Target age

■No limitations

□Limited (　　　Years ~　　　age)

③ Gender

□Male □Female ■Bisexual

(2) Eligibility criteria

Selection criteria: Patients who had ophthalmological images taken and health checkup participants

Exclusion criteria: Patients and health checkup participants who requested exclusion

11. Research period

Research period: After clinical research permission decision - until December 31, 2028

Registration period: January 1, 2002 to December 31, 2027

1 2. Research method

Collect ophthalmological images and videos taken during daily medical treatment or medical images taken during health checkups between January 1, 2002 and December 31, 2027 at data collection facilities including our university's ophthalmology department . A database is constructed by investigating gender, age, visual acuity, refraction, intraocular pressure, findings, diagnosis, and treatment details from medical records . Data is collected as needed, machine learning is performed each time, and the performance of the created algorithm or provided algorithm (Canon's AI approved by other countries) is analyzed. Many database creation studies like this one have been criticized for ceasing activity as soon as the public research funding required for creation ends, resulting in a waste of public tax money . However, we believe that it is desirable to continue collecting and machine learning while updating data storage formats and ethical standards, contributing to medical care in the areas where data is collected .

January 1, 2002 Permit date 2 February 3 , 2002 January 1 , 2002

photograph

Data collection ↓↓↓↓↓↓↓↓↓↓↓↓↓↓↓↓↓↓↓↓↓

Analysis ∨

∨

∨

December 31, 2027

Three analysis images. The images were taken during daily medical treatment unrelated to research. Data collection is carried out at any time after obtaining permission, so it is indicated with a ``↓''. The analysis performed on the permit date will be based on data from 2002 to the permit date, using only the second "→" . The analysis performed on the last day of data collection will be performed on the data from 2002 to that day, using the fourth "→". The analysis in between is performed using the information that is in the database at that time, using the third "→" symbol.

・The data collection facility will provide medical images and information to Jichi Medical University. Jichi Medical University will store its own facility data and the data provided by other facilities in a database managed by the Jichi Medical University Ophthalmology Department.

・The data analysis facility requests necessary data from Jichi Medical University, and Jichi Medical University outputs the requested data from the database and provides it electronically, and the data analysis facility uses the provided information to perform machine learning. , analyze the results, and verify the effectiveness and risks of AI using clinical data. Data, including those from overseas, will be sent as soon as the data is ready, and if a company is included in the data analysis facility, the dates will be limited in the joint research agreement so that the data will not be analyzed beyond the permitted schedule, and records of information sent will be kept. By retaining the data, it is possible to conduct follow-up tests and publish papers on the analysis results, ensuring transparency.

data flow

Data collection only facility

Jichi Medical University

Data collection and analysis facilities

Data analysis only facility

Regarding the above three types of facilities, the research costs required at each facility will be borne by each facility. When intellectual property or patents are generated in the future, researchers at the facility that developed them will be the inventors, but if the preprocessing of the data received by that facility includes the technology required for the patent, the processing The researchers at Jichi Medical University who came up with the idea and carried out the idea, or who came up with the idea and commissioned the data collection facility, are also considered inventors. In this case, the calculation of the proportionate distribution and contribution rate will be based on the proportion of costs incurred by each party, and will be determined through consultation and agreement between the developed facility and Jichi Medical University. Providing data alone does not constitute a contribution.

Other facilities ①: NitroSquare Co., Ltd., a ``software production'' company, produces software for machine learning, regardless of data analysis, and performs machine learning in collaboration with Jichi Medical University. Jichi Medical University's Department of Ophthalmology will pay 3 million yen for the research to NitroSquare Co., Ltd. , and NitroSquare Co., Ltd. will produce the software. In addition to the aforementioned expenses, NitroSquare Co., Ltd. is expected to pay 3 million yen out of pocket, but the details will be determined through consultation and agreement between Jichi Medical University Ophthalmology and NitroSquare Co., Ltd. Intellectual property rights related to inventions generated as a result of joint research shall be owned by Jichi Medical University.

Other facilities ②: "Practical" DeepEyeVision Co., Ltd. maintains research equipment regardless of data analysis, performs machine learning jointly with Jichi Medical University, and holds patent No. 6 745496 and other DeepEyeVision Co., Ltd. We will commercialize technology (know-how and algorithms) centered on patent rights and future patents expected from this research. After the inventions resulting from joint research ( excluding intellectual property rights such as patents held by DeepEyeVision Co., Ltd.) are put into practical use, a technology license fee will be paid to Jichi Medical University . Intellectual property rights related to inventions, etc. resulting from joint research will be shared, and the ownership ratio will be determined by agreement after consultation, depending on the degree of contribution to the invention, etc.

Please note that Canon Inc. only provides AI ( EyeArt ® : see attachment) that has been approved by other countries for trial purposes and does not conduct research.

・The cloud has an advantage in machine learning speed and cost over in-house computers, and only the necessary data is uploaded to the cloud and analyzed (although it is expressed as outsourcing, the outsourcing company only provides the cloud and does not handle the machine learning itself) (performed by each data analysis facility). The cloud service provider has obtained the certification listed in the subcontractor section, and guarantees security equivalent to or higher than that of storing data in Jichi Medical University's internal storage. The vendor only provides cloud storage, and the Jichi Medical University Department of Ophthalmology handles the storage work and the settings and encryption to prevent leakage).

- Comply with the provisions regarding the protection of personal information, etc. in the US HIPAA Act (Act on Health Insurance Portability and Accountability in the US: document used in deliberations within the Ministry of Health, Labor and Welfare attached).

・The principal investigator is a member of the Japan Society for Artificial Intelligence and will comply with the Society for Artificial Intelligence Ethics Guidelines (attached) not only for himself but also for the entire research.

13. Procedures for obtaining informed consent, etc.

(1) Collection of samples, information, etc.

□Use new samples, information, etc.

Its contents:

　　　　 □ Obtain written consent

□Obtain oral and recorded consent

Reasons and countermeasures:

□Guarantee the right to refuse information disclosure + opt-out

　　　　　　 Reasons and countermeasures:

　　　　　 □Others

　　　　　　 Reasons and countermeasures:

■Use existing samples, information, etc.

Contents: Ophthalmology images, videos, gender, age, visual acuity, refraction, intraocular pressure, findings, diagnosis, treatment details, below only at our university health checkup center, chest X-ray, mammography, CT (head, chest, abdomen), EGD (upper gastrointestinal endoscopy), abdominal ultrasound, mammary gland ultrasound, MDL (upper gastrointestinal barium contrast), ECG (electrocardiogram)

□Using samples obtained from the human body

□ Obtain written consent

□Obtain oral and recorded consent

□Guarantee the right to refuse information disclosure + opt-out

Reasons and countermeasures:

■Use information

□ Obtain written consent

□ Obtain verbal consent

■Guaranteeing the right to refuse by opting out

Reasons and countermeasures: Since the information falls under Guidelines ``Chapter 4, Section 8, Procedures for Obtaining Informed Consent, etc. 1 (2) A, C, and (5) C, Information Disclosure (attached). Consent will be obtained through opt-out, and the provision will be reported to the hospital director, etc. After this research is approved, it will be posted on the ophthalmology website, Saitama Medical Center website, Health Checkup Center website, and each joint research institution's website . Information disclosure documents will be posted on .

□Do not provide verbal explanations or provide explanatory documents.

Reasons and countermeasures:

(2) Requirements for representative, etc.

□Appoint a representative, etc.

<Policy for selecting proxy consenter, etc.>

① Types of research subjects and reasons

(a) Type

□ Minors (□ Under 16 years old □ Those who have completed a junior high school course, etc., or 16 years old or older)

□Adults who are objectively determined to lack the capacity to give informed consent.

□Dead person

□Others (　　　　　　　　　　　　　　　　　　　　　　　　　　　　　　)

(b) Reasons why it is necessary to include the person as a research subject:

② Type of representative consenter, etc.

□Parent □Spouse □Parent □Adult child

□Others (　　　　　　　　　　　　　　　　　　　)

③ Matters to be explained to the representative, etc.

□Same matters as explained to research subjects, etc.

□ Matters other than explanations to research subjects, etc.

Specific explanation:

■Do not have a representative , etc.

　　　　　<The reason>

■ This is not a research that requires a representative, etc.

　　　　　□ This is a research in which a representative, etc. will be appointed, but since all of the following apply, we will obtain the consent of the individual.

□ The research subjects are minors . However, it is judged that the person has completed a junior high school course, or is a minor over 16 years of age, and has sufficient decision-making ability regarding conducting research.

□ Non-invasive research

Disclose information about the implementation of the research, including the purpose of the research and the handling of samples and information, and provide parents or guardians of minors of research subjects with the opportunity to refuse the implementation or continuation of this research. Guaranteed

Specific measures:

(3) Procedures for obtaining informed assent

□Get informed assent

Explanation and method:

■Failure to obtain informed assent

■No need to obtain informed consent

□I need to obtain informed consent, but I cannot obtain it for the following reasons:

reason:

14. Adverse events and serious adverse events

(1) Definition of adverse events and serious adverse events

·none

(2) What to do if a serious adverse event occurs

・No adverse events

15. Ethical/Considerations, etc.

15.1 Regulations to be complied with

life science and medical research involving human subjects .

15.2 Handling of personal information, etc.

(1) Regarding samples and information to be collected

□Collect samples (blood, human tissue, etc.)

■Do not collect samples

■Collect information

■Medical information

□Questionnaire etc.

□Others ( )

□We do not collect information.

(2) Anonymization of samples, information, etc.

■Anonymize samples, information, etc.

□Anonymization (no correspondence table)

Timing of anonymization: □ At the start of the research □ At a certain time during the research period □ At the end of the research

How to anonymize:

■Anonymization (with correspondence table)

Timing of anonymization: ■At the start of the research □At a certain time during the research period □At the end of the research

Anonymization method: It is necessary to enter the date of medical treatment into the database because a difference of just one day can change visual acuity from 1.2 to blindness, but since the exact date of consultation can identify an individual, it is necessary to input the data first for each patient. By changing the measurement date to the 16th of each month and changing and outputting the dates of other data accordingly, you will no longer know what day the patient visited the hospital, even if you know the season of the data. Age is important for diagnosis and treatment, and the database must output the age at the time of imaging, but since date of birth can identify an individual, it is not output even if it is input. Only the age at the time of the photo is output. In the newborn period, the number of days after birth is important, so the number of days after birth is output. Since the output of the shooting date has been changed, the date of birth cannot be specified. Iris findings such as iris nodules and iris atrophy are important for diagnosis. Iris print images can become personal information if features are extracted for the purpose of personal identification, but since existing data for medical treatment purposes is used, there are no photographs that clearly show the entire iris that can be used for personal identification. Also, the health checkup center does not photograph the iris. If by any chance, some parts of the image will be masked so that it cannot be used for identification. Fundus photo file names, which often include each facility's patient ID and photographing date, are encrypted and processed. If the date or other information is reflected in the photo itself, it will be masked (software has already been developed). No personal information that can identify the patient is left in the data set that is actually analyzed. The database outputs only X-rays, mammography, CT (head, chest, abdomen), EGD (upper gastrointestinal endoscopy), abdominal ultrasound, mammary gland ultrasound, MDL (upper gastrointestinal barium angiography), and ECG (electrocardiogram). do. The correspondence table for patient ID anonymization is encrypted so that it can only be decrypted when the research director at each facility uses a password stored in a locked cabinet in each department at each facility. The IDs of only these patients will be known, and each facility will provide data to the university, but no correspondence tables will be submitted.

Reasons for creating a correspondence table

■After anonymization, the data may be corrected, changed, or added as necessary to ensure the accuracy, scientificity, and quality of the research, and will be traceable.

■When a subject requests withdrawal of consent or refusal to participate, it is necessary to identify the relevant data.

□Other ( please specify)

reason:

□Do not anonymize samples, information, etc.

Types of samples/information, etc.:

Reasons for not anonymizing:

(3) Joint use of samples, information, etc.

□No exchange of samples or information with joint research institutions

■ Exchanging samples and information with joint research institutions

■Domestic research institutions ■(Recipient institutions: Kagoshima University, Mie University, University of Yamanashi, National Institute of Informatics, NitroSquare Co., Ltd. , DeepEyeVision Co., Ltd., Tsukazaki Hospital, Hirosaki University)

(Persons responsible for the provision: Shozo Sonoda, Toru Ichio, Kenji Kashiwagi, Yu Kitsuregawa, Hideaki Imaizumi, Kei Shimada, Hidenori Takahashi, Hitoshi Tabuchi, Shinji Ueno)

■(Provided by: Jichi Medical University Saitama Medical Center, Aoki Eye Clinic, Okubo Eye Clinic, Saito Eye Clinic, Takahashi Eye Clinic, JCHO Tokyo Shinjuku Medical Center, Yokohama Minami Kyosai Hospital, Kagoshima University, Mie University, Yamanashi University, Shichiri Omiya Eye Clinic, Kikuna Yuda Eye Clinic, Heart Life Hospital, Kanazawa University, Tsukazaki Hospital, Omiya wa Mada Eye Clinic, Takada Contact Clinic, Hanyuta Eye Clinic, Hara Eye Clinic, Totsuka Eye Clinic, Ohanajaya Eye Clinic, Yokohama City University Citizens General Medical Center, Sanraku Hospital, Nihon University Hospital, Itabashi Hospital, Nihon University School of Medicine, Nagoya University, Osaka University, Hirosaki University)

(Persons responsible for the provision: Toshikatsu Kabuki, Shinsuke Aoki, Akira Okubo, Shinichiro Saito, Yuji Takahashi, Chihiro Mayama, Yasushi Ida, Shozo Sonoda, Toru Ichio, Kenji Kashiwagi, Kenichiro Yamazaki, Kentaro Yuda, Itaru Oyakawa, Kobayashi Ken, Hitoshi Tabuchi , Naoki Hamada , Yudai Motoyama, Naoto Hanyuta, Masa Hara, Nobuyoshi Fujioka, Yasuo Yanagi, Shinichi Nakamura, Koji Tanaka, Takahiko Hayashi, Shinji Ueno, Ryo Kawasaki, Shinji Ueno )

Items: Ophthalmology images, videos, gender, age, visual acuity, refraction, intraocular pressure, findings, diagnosis, treatment details, below only at our university health checkup center, chest X-ray, mammography, CT (head, chest, abdomen), EGD ( Upper gastrointestinal endoscopy), abdominal ultrasound, mammary gland ultrasound, MDL (upper gastrointestinal barium contrast), ECG (electrocardiogram)

Procedures for exchanging samples, information, etc. with other institutions:

After receiving permission to conduct research at our university, for institutions other than joint research institutions that have undergone comprehensive review at our university, joint researchers must submit the approved research plan of our university to the head of the joint research institution. Receive written permission to conduct research at the joint research institution in accordance with the university's research plan (research implementation permission letter from the head of the institution to the joint researcher). The database output information (table format and image files; correspondence tables, etc. are not included) will be encrypted and sent via the cloud (listed to the contractor), or as an email attachment if the size is small. Keep records of giving and receiving.

・How to manage correspondence tables, etc. and specific responses:

Patient ID correspondence tables, etc. are encrypted and decrypted only when the research director at each facility uses a password stored in a locked cabinet in each department at each facility. Only patients will be able to identify their IDs , and each collection facility will provide data to the university, and the university will provide data to each analysis facility, but no correspondence tables will be submitted.

■Overseas research institutions ■(Recipient institutions: National University of Singapore, The University of Tennessee, Stefan cel Mare University of Suceava, Federal University of Sao Paulo, University of Baghdad, Universiti Kebangsaan Malaysia)

　　　　　　　　　　　(Responsible person for the provision: Yasuo Yanagi, Siamak Yousefi・Alexandru Lavric・Rosen M. Hazarbassanov・Ali H.Al-Timemy・Zaid Alyasseri )

■(Provided by institutions: National University of Singapore, Federal University of Sao Paulo, The University of Brescia )

(Persons responsible for the provision: Yasuo Yanagi, Rosen M. Hazarbassanov , Vito Romano )

Items: Ophthalmology images, videos, gender, age, visual acuity, refraction, intraocular pressure, findings, diagnosis, treatment details, below only at our university health checkup center, chest X-ray, mammography, CT (head, chest, abdomen), EGD ( Upper gastrointestinal endoscopy), abdominal ultrasound, mammary gland ultrasound, MDL (upper gastrointestinal barium contrast), ECG (electrocardiogram)

Procedures for exchanging samples, information, etc. with other institutions:

The database output information (table format and image files; correspondence tables, etc. are not included) will be encrypted and sent via the cloud (listed to the contractor), or as an email attachment if the size is small. Keep records of giving and receiving.

Personal information is included in samples, information, etc. to be jointly used.

Contents: gender and age

Reason for using personal information: Gender and age are information used in daily medical practice to determine diagnosis and treatment policy, and it is thought that using artificial intelligence can more accurately assist in diagnosis and treatment policy decision-making.

15.3 Anticipated risks, benefits, and measures to minimize risks, etc.

(1) Burden and anticipated risks to research subjects

① Cost burden

□By participating in the research, research subjects will have to bear additional costs.

□Full amount to be paid by oneself (cost amount: approx.　　　　　circle)

□Partial self-pay (payment amount: approx.　　　　　circle)

□Within the scope of insurance treatment

□Others (　　　　　　　　　　　　　　)

■There is no additional cost burden for research subjects if they participate in the research.

② Other burdens and anticipated risks

□ Research subjects will have other burdens if they participate in the research.

Specific matters:

■ There are no other burdens on research subjects if they participate in the research.

(2) Benefits accruing to research subjects

① Reward to the target person

□Yes (specifically:　 　　　　　　　　　　　　　　　)

■None

② Other profits

　　　　　　□Yes Contents:

■None

(3) Comprehensive evaluation of the burden and expected risks and benefits that will occur to research subjects, and measures to minimize the burden and risk.

① Comprehensive evaluation

-No risk or profit.

② Measures to minimize burden and risk

·none.

③ Loss compensation

□There is compensation for losses incurred as a result of conducting this research.

Contents of compensation:

■There is no compensation for losses incurred as a result of conducting this research.

15.4 Handling of research results regarding research subjects

■There is no possibility of obtaining important knowledge regarding the health of research subjects or genetic characteristics that can be passed on to offspring.

□It is possible to obtain important knowledge regarding the health of research subjects and genetic characteristics that can be passed on to offspring.

Handling of research results:

15.5 Contents and method of reporting to the president (excluding reports of serious adverse events)

■Once a year , report the progress status of clinical research, etc., and the occurrence of adverse events, defects, etc. to the president without delay in the clinical research progress report ^*^

■ When clinical research, etc. is completed (cancelled) , promptly report to the president using a clinical research , etc. completion report ^*^

■If samples and information are provided to other institutions when clinical research is completed (cancelled), report to the university president without delay using a notification form regarding the provision of samples and information to other research institutions .

■ If you receive specimens or information from another institution when clinical research is completed (cancelled), report to the university president without delay using a notification form regarding receipt of specimens and information from other research institutions .

■ If records related to the exchange of samples and information are disposed of, promptly report to the university president using a sample and information disposal report.

1 5.6 Conflicts of interest related to research, such as funding sources for research, and conflicts of interest related to researchers' research, such as personal income, etc.

(1) Funding source

　　　 ■Course research expenses

　　　 □Entrusted research funding requester:

■Public research grant name: Fundamentals C General 21K09751 “Building a platform for elucidating the pathology of age-related macular degeneration through AI analysis of fundus images and cytokine concentrations (2021-2023) / Young 21K16903 “Predicting visual field test results using deep learning ”(2021-2022)

　　　 ■Other: Joint research funds

(2) Relationship with related organizations such as researchers

・There are no related organizations other than the joint research institutions and research outsourcing companies listed.

・There is no personal relationship (family relationship, advisor, etc.) between NitroSquare Inc. and the researcher. The company does not involve data analysis, but also software production and machine learning.

・Researcher Hidenori Takahashi is the founding shareholder and representative director of DeepEyeVision Inc. The company is not involved in data analysis, but also maintains research equipment and performs machine learning. The company is also considering the use or purchase of future patent-based technologies (know-how and algorithms) expected from this research, and is involved in the practical application of patent-based technologies.

・There is no personal relationship (family relationship, advisor, etc.) between Canon Inc. and the researcher. The company does not have a joint research agreement as it only provides AI ( EyeArt ® : see attachment) that has been approved in other countries for trial purposes. This AI is not provided specifically for this research, and may be provided to other research institutions as well. The person in charge is Ryuta Kikura, who is in charge of business planning.

(3) Conflict of interest situation

・Jichi Medical University Department of Ophthalmology and NitroSquare Co. , Ltd. will each pay 3 million yen per year for machine learning in joint research. Jichi Medical University's Ophthalmology Department's 3 million yen will be paid to NitroSquare Co., Ltd.

- In joint research between Jichi Medical University's Department of Ophthalmology and DeepEyeVision Co., Ltd., a portion of the labor costs for labeling will be paid by DeepEyeVision Co., Ltd. to Jichi Medical University, or labeling personnel will be dispatched. When using or purchasing future patent-based technology (know-how/algorithms) expected in this research, a royalty or compensation will be paid to Jichi Medical University from DeepEyeVision Inc.

(4) Benefits obtained from research

■Yes Institution contributing the economic benefit (company name, etc.): DeepEyeVision Co., Ltd.

Whose economic benefits belong to: □Individual researcher ■Course name, etc. ( ophthalmology )

■Others ( Jichi Medical University )

□None

(5) Patent rights, etc.

■Patent rights, etc. may arise.

Patent rights belong to: ■Jichi Medical University ■Individual researcher

■Others ( each joint research institution )

□There is no possibility of patent rights etc. arising

1 5.7 Method of disclosing information regarding research

( 1) Registration of research outline and results

□Register research outline and results

Registration location □Database maintained by the Ministry of Health, Labor and Welfare ( j RCT )

□National University Hospital Directors Conference ( U MIN )

　　 □Japan Medical Information Center, General Incorporated Association

□Japan Medical Association Public Interest Incorporated Foundation

■Do not register research outline and results

Specific reason: Because it is an observational study.

(2) Disclosure of personal data newly obtained through research (test results, etc.)

■ No new personal data will be obtained through this research.

□ There is new personal data obtained through this research.

① Disclosure to research subjects

□Disclose to research subjects

□Disclosed in principle □Disclosed to those who wish

□Do not disclose to research subjects

Reason for not disclosing:

② Disclosure to representative consenter

□Disclose to representative consenter

□Disclosure in principle □Disclosure subject to consent of research subjects □Disclosure to those who wish

□Do not disclose to proxy consenter

Reason for not disclosing:

□ There is no representative consent person in this research.

③ Disclosure to family members, etc.

□Disclose to family members, etc. (including bereaved family members)

□Disclosure in principle □Disclosure subject to consent of research subjects □Disclosure to those who wish

□Do not disclose to family members, etc. (including bereaved family members)

Reason for not disclosing:

(3) Publication of research results

■Publish research results

Publication method: ■ Paper presentation ■ Conference presentation ■ Internet publication

□Others (　　　　　　　　　　　　　　　　　　　　)

□Research results will not be made public

reason:

15.8 Handling of research in situations where research subjects are in immediate and clear danger to their lives

■Research is not conducted in situations where the research subject is in immediate and clear danger to life.

□Research is conducted in situations where the research subject is in immediate and clear danger to life.

□The research subject is in immediate and obvious life-threatening danger.

□This is an interventional study that cannot be expected to be sufficiently effective through normal medical treatment, and it is recognized that there is a good chance that the life-threatening situation of the research subject can be avoided by conducting the study.

□The burden and risks incurred by research subjects during the conduct of the research are the minimum necessary.

□Unable to immediately contact the representative consenter or the person who should become the representative consenter

15.9 Availability and content of compensation for health damage

■Research that does not involve invasion (no invasion or slight invasion) [Research not eligible for compensation]

□Research involving invasion (excluding minor invasion) [Research eligible for compensation]

□Compensation available

Specific details:

□No compensation

reason:

15.10 Responses regarding the provision of medical care after conducting research

■Research that does not go beyond normal medical treatment or does not involve medical treatment

□Research that involves medical procedures beyond normal medical treatment

Specific methods for providing medical care after conducting research:

16. Storage and disposal of samples, information, etc.

(1) Storage of samples and information during research and samples and information

① Types of samples, information, etc.

■Original samples/materials (case report forms, survey forms, etc.)

■Processed materials

□Agreement form

■Anonymization correspondence table

■Others (cipher key)

② Format of samples, information, etc.

□Paper media information

■Electronic information

□Sample

□Others ( )

③ Storage location

■Jichi Medical University (Specific location: Ophthalmology course medical office cabinet ■Can be locked □Cannot be locked)

■Joint research institution (specific location: Cabinet specified in each joint research facility defined in 2.3 ■Can be locked □Cannot be locked)

□Other (specific location:　　　　　　　　　　　　　□Can be locked □Cannot be locked)

(2) Storage of samples, information, etc. after research completion

□After the research is completed, samples, information, etc. will be stored for use for purposes other than the research purpose of this application.

Types of samples/information, etc.:

Reasons why storage is necessary:

□When using stored samples and information for other purposes, apply again to the Ethics Review Committee and obtain approval.

□When using stored samples, information, etc. for other purposes, obtain consent from research subjects again.

□When using stored samples, information, etc. for other purposes, do not obtain consent from research subjects again.

Reasons for not obtaining consent again:

Storage location

□Jichi Medical University (specific location:　　　　　　　　　□Can be locked □Cannot be locked)

□Joint research institute (specific location:　　　　　　　　　□Can be locked □Cannot be locked)

□Other (specific location:　　　　　　　　　　　　□Can be locked □Cannot be locked)

□After the research is completed, for a certain period of time (　　　　　Discard/discard after storing (months)

Types of samples/information, etc.:

Storage location

　　　　　□Jichi Medical University (specific location:　　　　　　　　　□Can be locked □Cannot be locked)

□Joint research institute (specific location:　　　　　　　　　□Can be locked □Cannot be locked)

□Other (specific location:　　　　　　　　　　　　□Can be locked □Cannot be locked)

□As this is an interventional study that involves invasion ( excluding minor invasion), 5 years have passed from the date on which the completion of the study was reported, or 3 years from the date on which the final publication of the results of the study was reported. It will be kept for a period of time until the later of 2018, and will be destroyed or disposed of after that period.

■ Immediately destroy/dispose of samples, information, etc. after the research is completed.

(3) How to destroy/dispose of samples, information, etc.

□Incineration after autoclave sterilization

□ Shred with a shredder

■Others ( Erase and discard data from storage using dedicated data erasing software )

(4) Storage of records regarding the exchange of samples, information, etc.

□No exchange of samples or information with other institutions

■Exchanging samples and information with other institutions

As this research involves the exchange of specimens and information with other institutions, records of the exchange of specimens and information will be kept until three years have passed from the day the specimens and information were provided .

■As the research is conducted by receiving samples and information from other institutions, records of the exchange of samples and information will be kept until five years have passed from the date of the report on the completion of the research .

(5) Report on storage and disposal methods of samples, information, etc.

■After the research is completed, if samples and information are being stored, report to the university president using a sample and information storage status report.

■If there is a change in the storage method for specimens, information, etc. after the research is completed, this will be reported to the university president using a specimen/information storage status change report.

■If samples, information, etc. are disposed of, report to the president using a sample/information disposal report.

17. Monitoring and auditing system and implementation procedures

17.1 Monitoring

　　　　■Do not carry out monitoring

　　　　□Carry out monitoring

　　　　Implementation structure and procedures:

17.2 Audit

　　　　■Do not conduct an audit

　　　　□Carry out an audit

　　　　Reasons for conducting an audit:

　　　　Implementation structure and procedures:

18.Consignment status of research-related work

□Do not outsource research-related work

■Outsourcing research-related work

■With contract (planned) □Without contract

Contents of commissioned work: Machine learning/data storage

Supervision method for subcontractors: Encryption is used to anonymize information so that no one knows who it belongs to, and correspondence tables (encryption keys) are not provided. Regularly check that the encryption has not been broken. If the measures are not complied with, the penalties will be raised until the measures are completed. We have obtained the certifications listed below to ensure confidentiality and protection of personal information, so a separate non-disclosure agreement is not required.

・ISO27001: International standard for information security. Evaluation targets: basic information security policy, organizational management, information management indicators, physical protection, communication protection, access control, etc. Update frequency: every year

Storage target: Ophthalmology images, videos, gender, age, visual acuity, refraction, intraocular pressure, findings, treatment details, below only at our university health checkup center, chest X-rays, mammography, CT (head, chest, abdomen), EGD (upper part) gastrointestinal endoscopy), abdominal ultrasound, mammary gland ultrasound, MDL (upper gastrointestinal barium contrast), ECG (electrocardiogram)

Storage method: Encrypted data

Responsible person/Storage location: Extreme -D Co., Ltd. Founder/Representative Director/CEO Naoki Shibata ( 2-2-25 Higashi-Shinagawa , Shinagawa-ku, Tokyo 140-0002 ) Sunwood Shinagawa Tennozu Tower 205 ), Amazon Web Services, Inc. ( https://aws.amazon.com ), Microsoft Azure ( https://azure.microsoft.com ), Google ( https://www.google) .co.jp ), Dropbox (https://dropbox.com) , slack (https://slack.com), box (https://box.com)

19. System and consultation desk where research subjects and their related parties can consult regarding research

(1) The necessity of genetic counseling and its system

·none

(2) Contact information

Affiliation: Ophthalmology

Title: Professor Name: Hidetoshi Kawashima

Phone number: 0285-58-7382

Internal phone number: 3526 PHS (if you have one): 7503

e-mail: hidemeak @ jichi.ac.jp

(3) Where to file complaints

Jichi Medical University Hospital Clinical Research Center Management Department (Tel: 0285-58-8933)
